# Clinical phenotypes of difficult-to-treat and mild asthma defined by cluster analysis

**DOI:** 10.1186/s12931-026-03740-0

**Published:** 2026-05-29

**Authors:** Mohammad Leily, Heena Mistry, Hongmei Zhang, Maria Larsson, Judit Varkonyi-Sepp, Ben Ainsworth, Liuyu Wei, Chellan Eames, J. J. Hudson-Colby, Adam Lewis, Anna Freeman, Hans Michael Haitchi, Paddy Dennison, Ratko Djukanovic, Sara Herrera-De La Mata, Grégory Seumois, Pandurangan Vijayanand, Mohammed A. Kyyaly, Elena Vorobeva, Elena Dabrio-Reina, Syed Hasan Arshad, Ramesh J. Kurukulaaratchy

**Affiliations:** 1 National Institute for Health Research (NIHR) Southampton Biomedical Research Centre at University Hospital Southampton NHS Foundation Trust, Southampton, UK; 2 Clinical and Experimental Sciences Department, Faculty of Medicine, University of Southampton, Southampton, UK; 3 Respiratory Medicine Department, University Hospital Southampton NHS Foundation Trust, Southampton, UK; 4 The David Hide Asthma & Allergy Centre, St Mary’s Hospital, Newport, Isle of Wight UK; 5 La Jolla Institute of Immunology, La Jolla, CA 92037 USA; 6 Division of Epidemiology, Biostatistics, and Environmental Health, School of Public Health, University of Memphis, Memphis, TN USA; 7 Clinical Health Psychology Department at the University Hospital Southampton NHS Foundation Trust, Southampton, UK; 8 School of Psychology, Faculty of Environmental and Life Sciences, University of Southampton, Southampton, UK; 9 Physiotherapy Department University Hospital Southampton NHS Foundation Trust, Southampton, UK; 10 School of Health Sciences, University of Southampton, Southampton, UK; 11 Institute for Life Sciences, University of Southampton, Southampton, UK; 12 Biological Sciences Graduate Program, University of California, San Diego, La Jolla, CA 92092 USA; 13 San Diego BioMed Research Institute, La Jolla, CA 92121 USA; 14 School of Technology and Maritime Industries, Southampton Solent University, Southampton, SO14 0YN UK; 15 Clinical Experimental Sciences, Southampton General Hospital, Mailpoint 810, F-Level, South Academic Block, Tremona Road, Southampton, Hampshire SO16 6YD UK

**Keywords:** Asthma, Cluster analysis, Comorbidities, Difficult-to-treat asthma, Mild asthma, Clinical phenotypes, Precision medicine, T2 inflammation

## Abstract

**Background:**

Cluster modelling has demonstrated asthma heterogeneity across disease severities, but contemporary data integrating difficult-to-treat and mild asthma with systematic assessment of comorbidity-focused treatable traits remain limited.

**Objective:**

To identify and characterise difficult-to-treat and mild asthma clusters in two UK cohorts: Wessex AsThma CoHort of Difficult Asthma (WATCH-DA) and a mild-asthma cohort from the Epigenetics of Severe Asthma study (EOSA-MA).

**Methods:**

Separate K-means clustering was applied to WATCH-DA (*n* = 498; 11 variables) and EOSA-MA (*n* = 67; 12 variables). Post-hoc comparisons evaluated demographic, inflammatory, physiological, comorbidity and patient-reported outcome profiles.

**Results:**

Six difficult-to-treat and two mild asthma clusters were identified respectively, all Type-2 (T2)-predominant. Difficult-to-treat asthma clusters differed by sex, age of asthma-onset, body mass index (BMI) and comorbidities. Two clinically controlled clusters, cluster-1 (Early-onset atopic controlled) and cluster-4 (Late-adult-onset non-atopic controlled), showed distinct comorbidity patterns despite lower overall morbidity. Three severe, exacerbation-prone, adult-onset, female predominant difficult-to-treat clusters (Adult-onset eosinophilic exacerbator [cluster-2], Young-adult-onset high-risk exacerbator [cluster-5], Adult-onset obese multimorbid symptomatic [cluster-6]) varied by blood eosinophil counts (BEC), spirometry, BMI, treatment needs, comorbidities, and quality of life. An Adolescent-onset obese atopic obstructive (cluster-3) showed fewer exacerbations but high BEC with worst spirometry and poor asthma control. In mild asthma, cluster-1 (Early-onset atopic mild) showed worse pathophysiological indices and asthma control than cluster-2 (Adolescent-onset mild) but similarly high comorbidity prevalence.

**Conclusion:**

Characterisation of difficult-to-treat and mild asthma clusters reveals diverse associated clinical traits and outcomes across the asthma severity spectrum. Recognition of these clusters and their associated comorbidities should prompt early personalised asthma management to address both airway-centric and comorbid disease aspects.

**Supplementary Information:**

The online version contains supplementary material available at 10.1186/s12931-026-03740-0.

## Background

Asthma is a chronic inflammatory airway disease characterised by variable airflow obstruction, bronchial hyperresponsiveness, and a broad range of clinical presentations. Despite structured guidelines, asthma still remains a clinical burden due to disease heterogeneity with substantial variation in clinical expression and natural history [1–3]. This heterogeneity spans age of asthma-onset, atopic status, physiological and inflammatory profiles, symptom burden, comorbidities and treatment response [4, 5]. 

To better capture this complexity, research has increasingly focused on asthma phenotypes, defined by particular observable clinical characteristics, and endotypes underpinned by distinct pathobiological mechanisms [6, 7]. Among various approaches, unsupervised cluster analysis has emerged as a useful method for identifying clinically relevant phenotypes without prior assumptions. Landmark cluster studies, have revealed reproducible severe asthma phenotypes, including early-onset allergic asthma, late-onset eosinophilic asthma, and obese non-eosinophilic asthma [8–13]. 

Previous cluster analyses have identified asthma phenotypes across a range of disease severities, including mild–moderate and refractory/severe asthma [8]. However, to our knowledge, no previous biologics-era cluster analysis has directly examined both well-characterised mild asthma and difficult-to-treat asthma cohorts with systematic integration of comorbidity-focused treatable traits. Including obesity [14], gastro-oesophageal reflux disease (GORD) [15], sinonasal disease [16], breathing pattern disorder [17], and psychological comorbidities [18] within clusters. Those comorbidities are increasingly recognised as important modifiers of asthma disease burden, adverse patient outcomes and treatment response, often within a complex framework of multimorbidity [19–21]. Accordingly, this study aimed to confirm and extend previous cluster-based asthma phenotyping by identifying and characterising clusters in contemporary difficult-to-treat (DA) and mild (MA) asthma cohorts using detailed clinical, physiological, inflammatory, patient-reported and comorbidity data.

The Wessex AsThma CoHort of Difficult Asthma (WATCH-DA) is a prospective, UK-based cohort of adults with DA (The Global Initiative for Asthma (GINA) treatment steps 4 or 5) [22]. With extensive characterisation data on asthma-focused clinical and pathophysiological parameters, and comorbidities. Conversely, a MA cohort from the Epigenetics of Severe Asthma study (EOSA-MA) (GINA treatment steps 1 or 2) provides a rare opportunity to evaluate data-driven clinical phenotyping in milder disease. The EOSA-MA cohort includes a subset of patients from the long-standing Isle of Wight Birth Cohort (IOWBC) [23], together enabling detailed clinical and biomarker characterisation.

The overall aim of this study is to enhance precision medicine in asthma care by identifying distinct DA and MA clinical phenotypes through assessment of their clinical, physiological, inflammatory and comorbidity profiles to inform future approaches to treatable trait-based stratification and management.

The four main objectives of this study were: (1) To identify DA and MA phenotypes in the respective WATCH-DA and EOSA-MA cohorts using K-means clustering; (2) To characterise the clinical, physiological, inflammatory, and comorbidity profiles across identified DA and MA clusters; (3) To compare the disease severity spectrum of asthma phenotypes across mild and difficult-to-treat asthma populations; (4) To potentially inform future approaches to treatable trait-based stratification and precision medicine in asthma care.

## Methods

### Study design and cohorts

This was a cross-sectional, observational study involving two independent UK-based asthma cohorts, the WATCH-DA and EOSA-MA cohorts, using comparable methodology for deeper clinical characterisation.

### WATCH difficult-to-treat asthma cohort

At the time of study, the WATCH-DA study recruited 501 adult DA patients from July 2015 to March 2019 from University Hospital Southampton NHS Foundation Trust (UHSFT) and the David Hide Asthma and Allergy Centre (DHAAC), Isle of Wight, UK. All patients were managed according to the BTS/SIGN step “high-dose therapies” and/or frequent or continuous oral corticosteroids (OCS) use (GINA treatment steps 4 and 5 equivalent) [3, 22]. Ethics approval and written informed consent from patients was obtained (REC reference: 14/WM/1226).

Briefly, comprehensive baseline characterisation was performed at enrolment including: demographics; asthma history; medication use; lung function tests; fractional exhaled nitric oxide (FeNO); blood biomarkers (BECs, blood neutrophils (BNC), total immunoglobulin E (IgE)); GINA Type-2 inflammation (T2)-high status - defined by either FeNO ≥ 20 ppb, BEC ≥ 0.2 × 10⁹/L (threshold chosen due to laboratory reporting to one decimal place), maintenance OCS (mOCS) use, or clinically allergen-driven disease (≥ 1 positive aeroallergen skin-prick test plus a relevant trigger), with sputum eosinophilia excluded due to limited samples); allergy skin prick testing (SPT) to 13 common aeroallergens; comorbidities defined using conventional clinical criteria (Supplementary Table 1); and health and disease-related questionnaires including Asthma Control Questionnaire-6 (ACQ-6), Nijmegen Score, Hospital Anxiety and Depression Scale (HADS), Sino-Nasal Outcome Test-22 (SNOT-22), Hull Cough Hypersensitivity Questionnaire, EuroQoL 5-Dimension 5-Level health today Visual Analogue Scale (Eq. 5D-5 L VAS), and St George’s Respiratory Questionnaire (SGRQ). Data were stored in a central WATCH-DA database housed at UHSFT and exported to SPSS v26 (IBM, NY, USA) The full study protocol is described elsewhere [23]. Three patients were excluded due to extreme outlier values for total IgE, leaving 498 patients for clustering analysis (Supplementary Fig. 1).

### EOSA mild asthma cohort

The EOSA-MA cohort included 69 participants with physician-diagnosed MA defined by BTS/SIGN treatment steps 1–2 (GINA steps 1–2) [3, 24], recruited from August 2018 to July 2019 as part of a larger NIH-funded EOSA study [24] (total *n* = 193). A subset (*n* = 23) was drawn from the IOWBC, a whole-population birth cohort initiated in 1989 to study the natural history of asthma and allergy, with detailed longitudinal data from birth to 26-years of age [25]. The remaining participants (*n* = 46) were identified from local IOW Allergy Clinics, primary care practices via review of electronic health records and community outreach (including use of the DHAAC website) (Supplementary Fig. 1). Ethics approval for the EOSA study was obtained (REC reference: 18/SC/0105). All participants provided written informed consent.

All participants underwent identical clinical, physiological, biomarker, comorbidity and questionnaire assessments to the WATCH-DA cohort. Data were stored securely in a DHAAC-based SPSS dataset. Two participants were excluded as outliers (one for extreme heavy smoking, one for an extreme outlier value for total IgE), leaving 67 participants for clustering analysis.

### Clustering variables and analysis

K-means cluster analysis was performed separately for each cohort using PROC FASTCLUS in SAS v9.4 (SAS Institute, Cary, NC, USA). The selection of clinical variables used for cluster analysis is detailed in Supplementary Methods. Categorical variables were transformed into numeric form prior to clustering. The number of clusters was determined using the Cubic Clustering Criterion (CCC), pseudo-F-statistic, and the R² statistic, in combination with clinical interpretability. Eleven clustering variables were used for WATCH-DA: age of asthma-onset, BMI, hospitalisations in past 12 months, Intensive Care Unit (ICU) admissions (ever), number of OCS courses in past 12 months, FeNO, post-bronchodilator (BD) Forced Expiratory Volume in 1 s (FEV₁) % predicted, BEC, serum Total IgE, ACQ-6 score, and sum of positive SPTs. Twelve clustering variables were used for EOSA-MA: age of asthma-onset, BMI, ICU admissions (ever), asthma-related GP visits in past 12 months, number of OCS courses in past 12 months, current inhaled corticosteroids (ICS) use, FeNO, pre-BD FEV₁ % predicted, BEC, serum total IgE, ACQ-6 score, and sum of positive SPTs. The differing and additional clinical variables used for the EOSA-MA cohort included GP visits in past 12 months (instead of hospitalisations in past 12 months) and current ICS use respectively to enable capturing the asthma severity spectrum in this population.

### Post-hoc trait analysis

Following cluster assignment, additional post-hoc analyses were conducted to evaluate differences between clusters in demographics, treatment, inflammatory (only a subset of WATCH-DA patients (*n* = 139) had induced sputum samples available for differential cell count analysis), physiological, and comorbidity profiles plus health outcomes.

### Statistical analysis

Cluster membership was used as the grouping variable for all post-hoc trait analyses. Categorical variables were represented as frequency; n (%), and compared across clusters using Chi-squared test, or Fisher’s exact test when expected cell counts < 5. Continuous variables were represented as mean (SD) or median (IQR), according to distribution. Between-cluster differences for continuous data were assessed using one-way ANOVA for normally-distributed variables and Kruskal–Wallis test for skewed variables. Where the omnibus test was significant, exploratory pairwise comparisons between clusters were made using independent-sample t-test or Mann–Whitney U test, as appropriate, to identify which clusters differed. A p-value < 0.05 was considered statistically significant. All post-hoc analyses were conducted using SPSS v26 (IBM, NY, USA).

## Results

### Difficult-to-treat asthma clusters

#### Main clustering outcomes

**Fig. 1:**
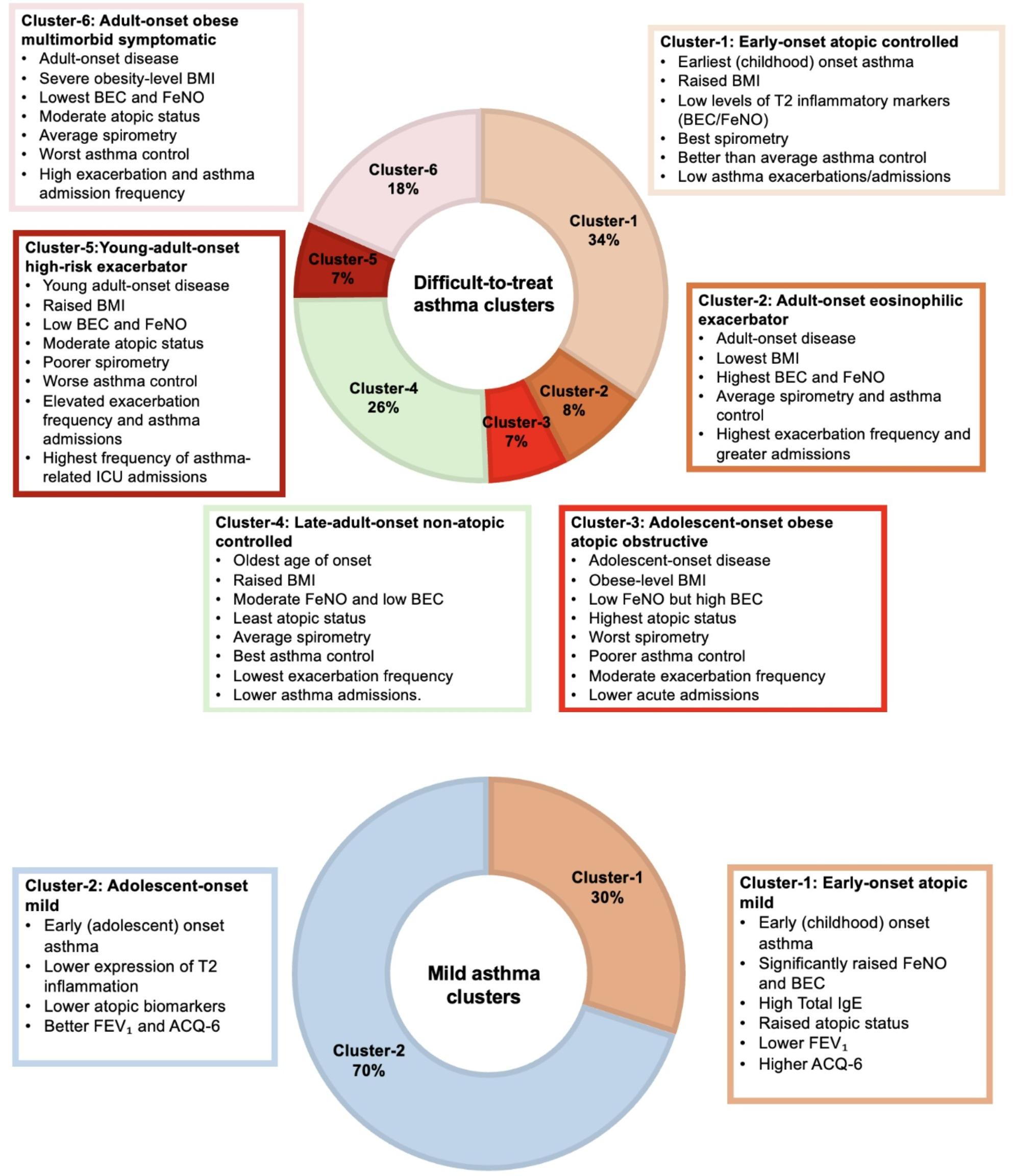
Summary characterisation of the 6 difficult-to-treat asthma clusters and 2 mild asthma clusters. Top: doughnut chart displaying the 6 difficult-to-treat asthma clusters according to cluster frequency. The boxes summarise each difficult-to-treat asthma cluster phenotype according to age of onset, BMI, morbidity, inflammatory and comorbidity profiles. The colours for each cluster depict the asthma severity spectrum; peach (cluster-1) and green (cluster-4) representing the lower disease severity clusters, whilst orange (cluster-2), red (cluster-3) and burgundy (cluster-5) highlight the more severe asthma spectrum clusters with increasing colour intensity. Pink (cluster-6) represents the more difficult-to-treat asthma cluster phenotype. Bottom: doughnut chart of the 2 mild asthma clusters according to cluster frequency. The boxes summarise each mild asthma cluster phenotype according to age of onset, BMI, morbidity, inflammatory and comorbidity profiles. Abbreviations: BMI, body mass index; T2, type-2; BEC, blood eosinophil count; FeNO, fractional exhaled nitric oxide; ICU, Intensive Care Unit; IgE, immunoglobulin E; FEV1, forced expiratory volume in 1 second; ACQ-6, Asthma Control Questionnaire-6

Cluster analysis using 11 selected variables identified six discrete DA clusters. A six-cluster solution was selected because, across candidate models with differing numbers of clusters, this showed the most favourable combination of cubic clustering criterion and pseudo-F statistics together with a high between-cluster R², whilst also yielding clinically coherent, non-redundant phenotypes (as detailed in Methods). All clustering variables showed significant differences across these clusters (Table 1). Resulting clustering variable-based characterisation is summarised below and in Fig. 1.

**Table 1:** Summary characterisation of clustering variables for the WATCH difficult-to-treat asthma cohort

Clustering Variables	Total Cohort	Cluster 1	Cluster 2	Cluster 3	Cluster 4	Cluster 5	Cluster 6	*P*-value
Number of subjects, % *(N*)	498	34.3 (171)	8.0 (40)	7.0 (35)	25.5 (127)	6.6 (33)	18.5 (92)	
Body mass index, kg/m^2^ * Missing*	30.9 (7.3) 6	29.4 (5.9) 2	***28.2*** (5.4) 0	32.0 (7.5) 1	28.3 (4.9) 1	28.8 (7.4) 0	**39.1** (7.2) 2	**< 0.0001**
Age of asthma onset, years * Missing*	23.7 (20.4) 22	***9.0*** (9.1) 6	28.6 ± 20.9 0	15.1 (16.0) 3	**47.1** (13.3) 9	18.5 (18.3) 0	22.8 (16.7) 4	**< 0.0001**
Hospitalisations past 12-months * Missing*	0.7 (1.7) 4	***0.3*** (0.7) 2	0.70 (1.5) 0	0.4 (0.8) 1	***0.3*** (0.8) 0	1.6 (2.1) 0	**2.0** (2.8) 1	**< 0.0001**
OCS courses past 12-months * Missing*	3.5 (3.5) 51	2.1 (2.0) 19	**6.4** (4.4) 3	4.7 (4.1) 3	*1.9* (1.9) 14	4.6 (4.2) 1	6.2 (3.8) 11	**< 0.0001**
Asthma-related ICU visits ever * Missing*	0.8 (1.9) 1	0.4 (0.9) 1	0.9 (1.3) 0	0.3 (0.7) 0	***0.2*** (0.5) 0	**6.5** (3.1) 0	0.5 (0.8) 0	**< 0.0001**
FeNO, ppb * Missing*	30.6 (34.0) 111	21.1 (19.3) 45	**103.5** (54.0) 6	21.7 (19.5) 9	32.2 (24.1) 33	23.8 (19.1) 2	***17.7*** (16.1) 16	**< 0.0001**
Blood eosinophil count, x 10^9^/L * Missing*	0.26 (0.33) 46	0.19 (0.15) 19	**0.88** (0.74) 5	0.41 (0.30) 6	0.21 (0.20) 10	0.20 (0.16) 1	***0.18*** (0.19) 5	**< 0.0001**
Total IgE, IU/L * Missing*	298.2 (595.4) 117	214.1 (278.6) 39	222.4 (311.5) 7	**2394.7** (922.3) 15	185.5 (319.8) 38	172.0 (210.2) 5	***111.6*** (148.4) 13	**< 0.0001**
Atopy status								
Number of positive SPT * Missing*	2.9 (3.0) 110	4.0 (3.2) 31	2.7 (3.1) 6	**4.7** (3.5) 4	***1.3*** (1.8) 33	2.2 (2.5) 9	2.1 (2.4) 27	**0.0010**
Subjects with ≥ 1 positive SPT, % *(N)* * Missing*	68.3 (265) 110	82.1 (115) 31	64.7 (22) 6	**83.9** (26) 4	***51.1*** (48) 33	62.5 (15) 9	60.0 (39) 27	**< 0.0001**
Clinic (post-BD) FEV_1_% predicted * Missing*	75.5 (22.9) 160	**78.4** (22.2) 57	75.1 (16.4) 15	***61.9*** (21.6) 10	76.5 (23.8) 37	70.5 (28.0) 7	76.6 (21.6) 34	**0.0300**
ACQ-6 score * Missing*	2.50 (1.35) 34	2.15 (1.12) 7	2.35 (1.43) 2	2.84 (1.28) 4	***1.83*** (1.22) 12	3.23 (1.11) 2	**3.74** (1.04) 7	**< 0.0001**

Categorical variables are represented as percentages (%) and frequencies (N). Continuous variables are shown as mean (SD). The number of missing values is included. One-way analysis of variance (ANOVA) was used to derive significance values for continuous variables between the 6 clusters. P-values <0.05 are statistically significant (in bold). Significantly different clusters are highlighted in bold (highest) and bold italics (lowest)

*Abbreviations: OCS *oral corticosteroids, *ICU *intensive care unit, *FeNO *fractional exhaled nitric oxide, *IgE *immunoglobulin E, *SPT *skin prick test, *BD *bronchodilator, *FEV *_*1*_forced expiratory volume in 1 second, *ACQ-6 *asthma control questionnaire-6

- Cluster-1 (*n* = 171; 34.3%); *Early-onset atopic controlled*: earliest childhood-onset disease, raised BMI, low BEC and FeNO, best spirometry, better-than-average asthma control, and low exacerbation and asthma-related admission frequency.
- Cluster-2 (*n* = 40; 8.0%); *Adult-onset eosinophilic exacerbator*: adult-onset disease, lowest BMI, highest BEC and FeNO, average spirometry and asthma control, but the highest exacerbation frequency and asthma-related admissions.
- Cluster-3 (*n* = 35; 7.0%); *Adolescent-onset obese atopic obstructive*: adolescent-onset disease, obese-level BMI, low FeNO but high BEC, highest atopy, worst spirometry, poorer asthma control, moderate exacerbation frequency and lower acute admission frequency.
- Cluster-4 (*n* = 127; 25.5%); *Late-adult*-*onset non-atopic controlled*: oldest age of asthma onset, raised BMI, moderate FeNO, low BEC, least atopy, average spirometry, best asthma control, lowest exacerbation frequency and lower admission frequency.
- Cluster-5 (*n* = 33; 6.6%); *Young-adult-onset high-risk exacerbator*: young-adult-onset disease, raised BMI, low BEC and FeNO, moderate atopy, poorer spirometry, worse asthma control, elevated exacerbation and admission frequency, and the highest frequency of asthma-related ICU admissions.
- Cluster-6 (*n* = 92; 18.5%); *Adult-onset obese multimorbid symptomatic*: adult-onset disease, obesity-level BMI, lowest BEC and FeNO, moderate atopy, average spirometry, worst asthma control, and high exacerbation and admission frequency.

#### Post-hoc cluster characterisation

**Fig. 2:**
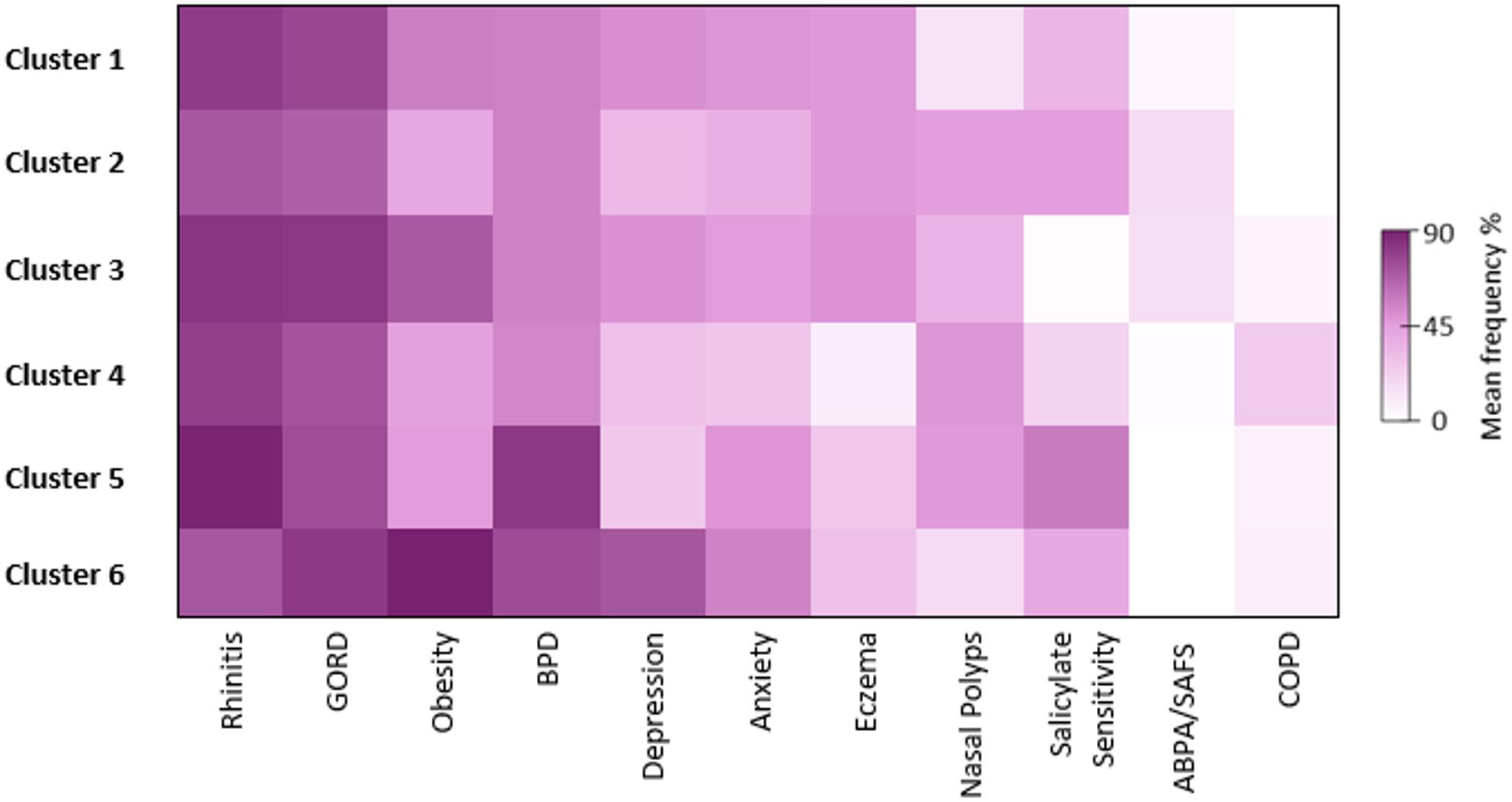
Comorbidity characteristics of the 6 difficult-to-treat asthma cluster phenotypes. Heatmap displaying the mean frequency of comorbidities for each difficult-to-treat asthma cluster. All comorbidities except for rhinitis and GORD were statistically significant (*P*<0.05; see Table 3). Abbreviations: GORD, gastro-oesophageal reflux disease; BPD, breathing pattern disorder; ABPA/SAFS, allergic bronchopulmonary aspergillosis/severe asthma with fungal sensitisation; COPD, chronic obstructive pulmonary disease

Baseline clinical characteristics and inflammatory profiles are shown in Table 2. Reported comorbidities, health-related quality of life and disease-related questionnaire outcomes are shown in Table 3, with key comorbidity patterns summarised graphically in Fig. 2. Lung function measurements are presented in Supplementary Table 2. Summary characterisation of the clusters is displayed in Fig. 1.

**Table 2:** Baseline clinical characteristics and inflammatory profiles of the WATCH difficult-to-treat asthma clusters

	Total Cohort	Cluster 1	Cluster 2	Cluster 3	Cluster 4	Cluster 5	Cluster 6	*P*-value
	Number of subjects, % (*N*)	498	34.3 (171)	8.0 (40)	7.0 (35)	25.5 (127)	6.6 (33)	18.5 (92)
Sex, % female *(N*)	65.3 (325)	74.3 (127)	60.0 (24)	***48.6*** (17)	48.8 (62)	69.7 (23)	**78.3** (72)	**< 0.0001**
Current age, years	50.8 (15.7)	***45.1*** (15.3)	50.5 (18.1)	49.3 (14.8)	**61.6** (11.0)	48.8 (16.6)	48.0 (13.6)	**< 0.0001**
Smoking status, % (*N*)								
Never	52.3(260)	**63.2** **(108)**	62.5(25)	60(21)	***40.2*** ***(51)***	51.5(17)	41.3(38)	**< 0.0001**
Current	5.6(28)	2.9(5)	***0*** ***(0)***	2.9(1)	5.5(7)	9.1(3)	**13.0** **(12)**	
Ex	42.1(209)	***33.9*** ***(58)***	37.5(15)	37.1(13)	**53.5** **(68)**	39.4(13)	45.7(42)	
* Missing*	1	0	0	0	1	0	0	
Healthcare Utilisation								
Intubated ever, % * (N)*	13.5 (67)	8.2 (14)	22.5 (9)	11.4 (4)	***4.7*** (6)	**63.6** (21)	14.1 (13)	**< 0.0001**
* Missing*	2	2	0	0	0	0	0	
Days lost from work past 12 months, % * (N)*	23.4 (48.9)	18.0 (41.3)	22.1 (67.5)	***12.5*** (25.5)	15.9 (47.3)	**55.0** (39.1)	50.9 (57.3)	**0.005**
* Missing*	230	66	10	19	65	15	55	
Medication								
Inhaled corticosteroids dose, µg BDP equiv.	2588.6 (986.6)	2687.4 (997.1)	2573.6 (850.6)	2950.2 (935.5)	2420.0 (957.8)	2572.9 (1050.9)	2515.6 (1039.8)	0.919
* Missing*	89	32	4	6	19	5	23	
Maintenance OCS use, % (*N*)	30.0 (143)	23.4 (44)	42.5 (17)	25.0 (8)	27.2 (34)	34.4 (11)	32.6 (29)	0.448
* Missing*	22	13	0	3	2	1	3	
Macrolide use, % (*N*)	20.5 (102)	18.1 (31)	15.0 (6)	31.4 (11)	17.3 (22)	30.3 (10)	23.9 (22)	0.200
Itraconazole use, % (*N*)	3.6 (18)	3.5 (6)	2.5 (1)	**14.3** (5)	3.1 (4)	6.1 (2)	***0***	**0.008**
Biologics at enrolment, % (*N*)	17.1 (85)	23.4 (40)	15.0 (6)	17.1 (6)	12.6 (16)	**24.2 (8)**	***9.8 (9)***	**0.044**
Omalizumab at enrolment, % (*N*)	11.6 (58)	18.1 (31)	10.0 (4)	11.4 (4)	***4.7 (6)***	**18.2 (6)**	7.6 (7)	**0.007**
Mepolizumab at enrolment, % (*N*)	5.2 (26)	4.7 (8)	5.0 (2)	5.7 (2)	7.9 (10)	6.1 (2)	2.2 (2)	0.593
Biologics started post-enrolment, % (*N*)	24.3 (121)	***17.5 (30)***	32.5 (13)	**37.1 (13)**	20.5 (26)	36.4 (12)	29.3 (27)	**0.017**

**Baseline inflammatory profiles**	**Total Cohort**	**Cluster 1**	**Cluster 2**	**Cluster 3**	**Cluster 4**	**Cluster 5**	**Cluster 6**	***P*** **-value**
Blood/Serum								
Eosinophil count ≥ 0.2 × 10^9^/L, *% (N*)	55.3 (250)	52.0 (79)	**85.7 (30)**	82.8 (24)	52.1 (61)	50.0 (16)	***46.0 (40)***	**< 0.0001**
* Missing*	46	19	5	6	10	1	5	
Max. Eosinophil count, x 10^9^/L	0.64 (0.66)	0.54 (0.59)	**1.15** (0.80)	0.80 (0.44)	0.71 (0.82)	0.57 (0.57)	***0.47*** (0.40)	**< 0.0001**
* Missing*	11	5	1	3	2	0	0	
Neutrophil count, x 10^9^/L	6.27 (2.85)	6.1 (2.9)	5.8 (1.8)	***5.5*** (1.9)	6.1 (2.9)	5.7 (1.8)	**7.5** (3.3)	**< 0.0001**
* Missing*	44	20	5	4	9	1	5	
Aspergillus fumigatus-specific IgE, IU/L	1.46 (7.39)	0.88 (3.60)	3.15 (12.20)	**6.34** (12.25)	1.67 (9.99)	0.12 (0.43)	***0.08*** (0.65)	**0.001**
* Missing*	63	22	1	5	19	6	10	
Airway								
FeNO ≥ 20 ppb, % (*N*)	48.8 (189)	41.3 (52)	**97.1 (33)**	38.5 (10)	60.6 (57)	48.4 (15)	***28.9 (22)***	**< 0.0001**
* Missing*	111	45	6	9	33	2	16	
Sputum Eosinophils, %	8.5 (14.1)	***2.26*** (3.44)	**22.12** (21.09)	10.91 (11.06)	11.43 (15.47)	15.62 (25.60)	2.27 (4.29)	**< 0.0001**
* Missing*	359	132	28	22	81	26	70	
Sputum Eosinophils ≥ 2%	47.5 (66)	***30.8*** (12)	**75.0** (9)	61.5 (8)	56.5 (26)	42.9 (3)	36.4 (8)	**0.039**
Sputum Neutrophils, %	42.85 (24.84)	45.99 (25.46)	38.46 (25.94)	42.83 (25.91)	43.00 (23.63)	34.61 (21.65)	41.98 (27.62)	0.881
* Missing*	359	132	28	22	81	26	70	
Sputum Neutrophils ≥ 61%	27.3 (38)	33.3 (13)	16.7 (2)	30.8 (4)	28.3 (13)	0	27.3 (6)	0.532
Sputum Inflammatory Phenotype, *% (N*)								
Eosinophilic	36.7 (51)	20.5 (8)	66.7 (8)	53.8 (7)	45.7 (21)	42.9 (3)	18.2 (4)	0.068
Neutrophilic	16.5 (23)	23.1 (9)	8.3 (1)	23.1 (3)	17.4 (8)	0 (0)	9.1 (2)	
Mixed granulocytic	10.8 (15)	10.3 (4)	8.3 (1)	7.7 (1)	10.9 (5)	0 (0)	18.2 (4)	
Paucigranulocytic	36.0 (50)	46.2 (18)	16.7 (2)	15.4 (2)	26.1 (12)	57.1 (4)	54.5 (12)	
GINA T2-high status, % (*N*)	93.5 (435)	95.6 (151)	**100.0 (40)**	**100.0 (31)**	91.5 (108)	90.0 (30)	***88.2 (75)***	**0.049**
* Missing*	33	13	0	4	9	0	7	

Categorical variables are represented as percentages (%) and frequencies (N), and continuous variables as mean (SD). The number of missing values is included. Chi-squared test was applied for categorical variables and one-way ANOVA was used to derive significance values for continuous variables between the 6 clusters. P-values <0.05 are statistically significant (in bold). Significantly different clusters are highlighted in bold (highest) and bold italics (lowest)

Biologics include Mepolizumab (anti-IL-5); Omalizumab (anti-IgE)

*Abbreviations: BDP equiv. *beclomethasone dipropionate equivalent/day, *OCS *oral corticosteroids, *Max. *maximum, *IgE *immunoglobulin E, *FeNO *fractional exhaled nitric oxide, *ppb *parts per billion, *GINA *Global Initiative for Asthma, *T2 *type-2

**Table 3:** Reported comorbidities plus health and disease-related questionnaires in the WATCH difficult-to-treat asthma clusters

	Total Cohort	Cluster 1	Cluster 2	Cluster 3	Cluster 4	Cluster 5	Cluster 6	*P*-value
	Comorbidities % (*N*)							
Rhinitis	67.6 (300)	70.3 (109)	59.5 (22)	72.4 (21)	68.7 (79)	79.2 (19)	59.5 (50)	0.440
* Missing*	54	16	3	6	12	9	8	
Gastro-oesophageal reflux disease	65.1 (315)	65.9 (110)	56.4 (22)	71.9 (23)	61.0 (75)	63.6 (21)	71.1 (64)	0.614
* Missing*	14	4	1	3	4	0	2	
Obesity (BMI ≥ 30)	48.6 (239)	44.4 (75)	***30.0*** (12)	58.8 (20)	31.7 (40)	33.3 (11)	**90.0** (81)	**< 0.0001**
* Missing*	6	2	0	1	1	0	2	
Breathing Pattern Disorder	48.6 (230)	43.7 (69)	43.2 (16)	42.9 (15)	***41.5*** (51)	**71.0** (22)	64.0 (57)	**0.001**
* Missing*	25	13	3	0	4	2	3	
Inducible laryngeal obstruction	14.6 (65)	14.1 (21)	8.3 (3)	9.1 (3)	11.6 (13)	20.0 (6)	22.6 (19)	0.168
* Missing*	54	22	4	2	15	3	8	
Depression	36.5 (165)	38.2 (58)	25.0 (9)	37.5 (12)	23.3 (28)	***20.8*** (5)	**60.2** (53)	**< 0.0001**
* Missing*	46	19	4	3	7	9	4	
Anxiety	32.7 (147)	36.2 (55)	27.8 (10)	33.3 (10)	***21.7*** (26)	36.0 (9)	**43.0** (37)	**0.033**
* Missing*	49	19	4	5	7	8	6	
Eczema ever	25.8 (127)	34.9 (59)	35.0 (14)	**37.1** (13)	***10.5*** (13)	21.2 (7)	23.1 (21)	**< 0.0001**
* Missing*	6	2	0	0	3	0	1	
Nasal polyps ever	23.4 (109)	***13.4*** (21)	32.5 (13)	26.7 (8)	**35.6** (42)	34.4 (11)	15.7 (14)	**< 0.0001**
* Missing*	32	14	0	5	9	1	3	
Salicylate sensitivity ever	25.0 (123)	26.2 (44)	32.5 (13)	***5.9*** (2)	17.5 (22)	**45.5** (15)	29.7 (27)	**0.012**
* Missing*	6	3	0	1	1	0	1	
Sulphite sensitivity ever	7.8 (38)	6.6 (11)	12.5 (5)	5.7 (2)	7.2 (9)	12.1 (4)	7.7 (7)	0.934
* Missing*	8	5	0	0	2	0	1	
ABPA/SAFS ever	6.7 (33)	7.7 (13)	**15.0** (6)	14.3 (5)	5.6 (7)	3.0 (1)	***1.1*** (1)	**0.047**
* Missing*	8	3	0	0	2	0	3	
Non-CF bronchiectasis ever	14.2 (70)	10.7 (18)	30.0 (12)	20.0 (7)	15.2 (19)	12.1 (4)	11.0 (10)	0.178
* Missing*	6	3	0	0	2	0	1	
COPD ever	10.1 (50)	***4.8*** (8)	5.0 (2)	8.6 (3)	**19.8** (25)	9.1 (3)	9.9 (9)	**0.023**
* Missing*	5	3	0	0	1	0	1	

	Health and Disease-related Questionnaires							
	Total Cohort	Cluster 1	Cluster 2	Cluster 3	Cluster 4	Cluster 5	Cluster 6	*P*-value
ACQ6 score ≥ 1.50, % (*N*)	76.9 (357)	72.6 (119)	73.7 (28)	90.3 (28)	***58.3 (67)***	96.8 (30)	**100.0 (85)**	**< 0.0001**
* Missing*	34	7	2	4	12	2	7	
SNOT-22 score	35.1 (21.4)	31.6 (20.1)	35.3 (21.6)	34.8 (23.8)	***31.4*** (18.7)	42.5 (26.9)	**44.9** (20.9)	**0.001**
* Missing*	177	48	12	13	55	11	38	
Hull cough hypersensitivity score	26.2 (15.6)	***24*** (14.8)	26.6 (17.2)	24.7 (18.0)	24.2 (16.2)	30.0 (14.8)	**32.0** (13.9)	**0.008**
* Missing*	123	39	7	10	38	5	24	
Hull cough hypersensitivity score > 13, % (*N*)	77.3 (290)	75.0 (99)	69.7 (23)	***68.0*** (17)	71.9 (64)	**92.9** (26)	89.7 (61)	**0.015**
Nijmegen score	22.1 (12.4)	21.7 (10.6)	***17.2*** (12.0)	21.0 (13.8)	19.2 (12.4)	26.1 (13.7)	**28.4** (12.4)	**< 0.0001**
* Missing*	127	38	10	10	35	7	27	
Nijmegen Score > 23, *%* (*N*)	42.0 (156)	42.1 (56)	36.7 (11)	40.0 (10)	***29.3*** (27)	53.8 (14)	**58.5** (38)	**0.010**
HADS-D score	5.4 (4.4)	4.7 (4.0)	4.9 (4.4)	5.4 (3.4)	***4.4*** (3.6)	5.8 (4.8)	**8.2** (5.0)	**< 0.0001**
* Missing*	75	28	4	8	16	6	13	
HADS-D Score ≥ 11, *%* (*N*)	14.9 (63)	12.6 (18)	16.7 (6)	***7.4*** (2)	8.1 (9)	22.2 (6)	**27.8** (22)	**0.004**
HADS-A score	7.0 (4.7)	7.2 (4.7)	***5.9*** (4.1)	7.0 (4.7)	6.0 (4.2)	6.5 (5.6)	**8.8** (5.1)	**0.002**
* Missing*	76	28	5	7	19	6	11	
HADS-A Score ≥ 11, *%* (*N*)	21.8 (92)	24.5 (35)	14.3 (5)	28.6 (8)	***11.1*** (12)	18.5 (5)	**33.3** (27)	**0.006**
Equation 5D-5 L Health Today VAS	64.0 (20.3)	68.2 (20.1)	**73.9** (15.1)	56.5 (13.4)	64.4 (21.9)	62.5 (20.2)	***53.8*** (18.9)	**0.012**
* Missing*	334	114	27	22	81	25	65	
SGRQ total score	50.7 (21.0)	43.6 (18.4)	50.9 (21.8)	57.1 (17.6)	***38.8*** (21.1)	54.0 (18.9)	**69.1** (14.3)	**< 0.0001**
* Missing*	120	33	7	9	44	7	20	

Comorbidities are represented as percentages (%), frequency (*N*) and the number of missing values. Chi-squared test was applied for categorical variables. P-values <0.05 are statistically significant (in bold). Significantly different clusters are highlighted in bold (highest) and bold italics (lowest)

*Abbreviations: BMI *body mass index, *ABPA/SAFS *allergic bronchopulmonary aspergillosis/severe asthma with fungal sensitisation, *CF *cystic fibrosis, *COPD *chronic obstructive pulmonary disease, *ACQ-6 *Asthma Questionnaire Score-6, *SNOT-22 *Sino-Nasal Outcome Test-22, *HADS-D *Hospital Anxiety and Depression scale-Depression component, *HADS-A *Hospital Anxiety and Depression scale-Anxiety component, *EQ5D-5L Health Today VAS *EuroQoL 5-Dimension 5-Level Health Today Visual Analogue Scale, *SGRQ *St Georges Respiratory Questionnaire

- Cluster-1: *Early-onset atopic controlled*. This was the largest cluster and showed female predominance, the youngest current age, earliest childhood asthma onset, and a mostly never-smoking profile. It had low FeNO and BEC, low exacerbation and asthma-related admission frequency, and the best spirometry profile. Although rhinitis, GORD and eczema were common, this cluster had the lowest nasal polyp and COPD prevalence. Questionnaire outcomes were comparatively favourable, including lower Hull cough hypersensitivity scores and better asthma control than most other clusters.
- Cluster-2: *Adult-onset eosinophilic exacerbator*. This was a smaller adult-onset cluster with female predominance, the lowest BMI, highest FeNO and BEC, and universal GINA-defined T2-high status. It had the highest exacerbation frequency and asthma-related admissions, despite average spirometry and asthma control. In the induced sputum subset, sputum eosinophils and the proportion with sputum eosinophils ≥ 2% were highest in this cluster. Comorbidity burden was generally lower than in several other clusters, with the lowest obesity prevalence and relatively low psychological comorbidity.
- Cluster-3: *Adolescent-onset obese atopic obstructive*. This cluster had adolescent-onset disease, obesity-level BMI, high BEC despite low FeNO, and the highest atopic burden. It showed the worst spirometry, including lower FEV₁ % predicted, FVC % predicted, FEV₁/FVC ratio and FEF₂₅–₇₅, together with the highest RV/TLC ratio. Clinically, it had poorer asthma control but only moderate exacerbation frequency and lower acute admission frequency. It also showed the highest Aspergillus fumigatus-specific IgE, highest ABPA/SAFS prevalence and highest eczema prevalence, with greater post-enrolment biologics initiation and itraconazole use.
- Cluster-4: *Late-adult-onset non-atopic controlled*. This cluster had the oldest age of asthma onset, older current age, raised BMI, low BEC, moderate FeNO and the lowest atopic burden. It was enriched for ex-smokers and had the best asthma control, lowest exacerbation frequency and lower asthma-related admission frequency. Spirometry was broadly average, while COPD and nasal polyps were most prevalent in this cluster. Questionnaire outcomes were generally favourable, including the lowest SNOT-22 score, lowest proportion with Nijmegen score > 23, lowest HADS-D score, lowest anxiety prevalence and best SGRQ total score.
- Cluster-5: *Young-adult-onset high-risk exacerbator*. This was a small young-adult-onset cluster with female predominance, raised BMI, low FeNO and BEC, moderate atopy, poorer spirometry and worse asthma control. It showed elevated exacerbation and admission frequency, the highest frequency of asthma-related ICU admissions and intubation, and the greatest number of days lost from work. Biologics use at enrolment, omalizumab use and post-enrolment biologics initiation were highest or among the highest in this cluster. Comorbidity features included the highest breathing pattern disorder prevalence and highest salicylate sensitivity prevalence.
- Cluster-6: Adult-onset obese multimorbid symptomatic. This adult-onset cluster showed female predominance, obesity-level BMI, the lowest FeNO and BEC, moderate atopy, average spirometry, worst asthma control and high exacerbation and admission frequency. It had the highest obesity prevalence and the greatest psychological comorbidity burden, including the highest depression and anxiety prevalence. It also had the highest SNOT-22, Hull cough hypersensitivity, Nijmegen, HADS-D and HADS-A scores, with the worst EQ-5D-5 L health today VAS and SGRQ total score, indicating the greatest patient-reported symptom and quality-of-life burden.

### Mild asthma clusters

#### Main clustering outcomes

Cluster analysis with 12 selected variables identified two discrete MA clusters. A two-cluster solution was selected favouring clinically coherent, non-redundant MA phenotypes. Clustering variables with significant differences between clusters included BEC, total IgE, FeNO, FEV₁ % predicted, atopic status and ACQ-6 (Table 4). Resulting cluster-based characterisation is summarised below and in Fig. 1.

**Table 4:** Summary cluster characterisation, clinical characteristics, inflammatory profiles, comorbidities plus health and disease-related questionnaires outcomes in the NIH EOSA mild asthma clusters

	Total Cohort	Cluster 1	Cluster 2	*P*-value
Number of subjects, % (*N*)	67	29.9 (20)	70.1 (47)	
CLUSTERING VARIABLES				
Body mass index, kg/m^2^				
* Median (IQR)* * Min*, *Max*	26.3 (7.3) 16.5, 42.4	25.3 (4.0) 16.5, 38.5	26.4 (9.0) 20.5, 42.4	0.113
Age of asthma onset, years				
* Median (IQR)* * Min*,* Max* * Missing*	13.0 (22.0) 0, 60.0 2	8.0 (6.0) 3.0, 49.0 1	16.0 (27.0) 0, 60.0 1	0.293
Asthma-related GP visits past 12-months				
* Median (IQR)* * Min*,* Max*	0 (0) 0, 4.0	0 (0) 0, 2.0	0 (0) 0, 4.0	0.587
OCS courses past 12-months				
* Median (IQR)* * Min*,* Max*	0 (0) 0, 2.0	0 (0) 0, 2.0	0 (0) 0, 2.0	1
Asthma-related ICU visits ever				
* Median (IQR)* * Min*,* Max*	0 (0) 0, 2.0	0 (0) 0, 0	0 (0) 0, 2.0	0.575
Inhaled corticosteroids use, % (*N*)	58.2 (39)	65.0 (13)	55.3 (26)	0.591
FeNO, ppb				
* Median (IQR)* * Min*,* Max*	27.0 (27.0) 5.0, 124.0	49.0 (42.8) 24.0, 124.0	18.0 (17.0) 5.0, 91.0	**< 0.0001**
Blood eosinophil count, x 10^9^/L				
* Median (IQR)* * Min*,* Max*	0.20 (0.20) 0.00, 0.60	0.30 (0.28) 0.10, 0.60	0.10 (0.10) 0.00, 0.40	**< 0.0001**
Total IgE, IU/L				
* Median (IQR)* * Min*,* Max* * Missing*	78.0 (221.00) 1.0, 2667.0 3	420.0 (681.0) 46.0, 2667.0 1	62.0 (120.0) 1.0, 1712.0 2	**< 0.0001**
Pre-BD FEV_1_% predicted				
* Median (IQR)* * Min*,* Max*	92.0 (16.0) 62.0, 138.0	82.5 (25.3) 62.0, 138.0	95.0 (17.0) 63.0, 117.0	**0.006**
Atopy status				
Number of positive SPT				
* Median (IQR)* * Min*,* Max* * Missing*	3.0 (5.0) 0, 12.0 2	2.0 (4.0) 2.0, 10.0 0	2.0 (5.0) 0, 12.0 2	**< 0.0001**
* Subjects with ≥ 1 positive SPT, % (N)*	73.8 (48)	100.0 (20)	62.2 (28)	**0.001**
ACQ6 score				
* Median (IQR)* * Min*,* Max* * Missing*	0.58 (0.80) 0, 2.70 1	0.83 (0.80) 0.20, 2.30 1	0.33 (0.80) 0, 2.70 0	**0.001**
CLINICAL CHARACTERISATION				
Sex, % female *(N*)	60.9 (42)	40.0 (8)	72.3 (34)	**0.012**
Age at enrolment, years	38.3 (11.9)	38.4 (11.2)	38.3 (12.3)	0.824
Smoking Status, % (*N*)				
Never Current Ex	47.8 (32) 20.9 (14) 31.3 (21)	40.0 (8) 25.0 (5) 35.0 (7)	51.1 (24) 19.1 (9) 29.8 (14)	0.701
Asthma duration, years * Missing*	20.5 (12.4) 2	24.6 (10.0) 1	18.8 (13.0) 1	0.088
Healthcare Utilisation				
Intubated ever, % *(N)*	0 (0)	0 (0)	0 (0)	-
Hospitalisations past 12-months, % *(N)*	0 (0)	0 (0)	0 (0)	-
Days lost from work past 12-months, % *(N)* * Missing*	0.56 (2.5) 3	1.40 (4.31) 0	0.18 (0.62) 3	0.069
Medication				
Inhaled corticosteroid dose, µg BDP equiv.	757.1 (469.8)	663.5 (336.1)	803.9 (523.8)	0.386
SABA use, % (*N*)	89.6 (60)	85.0 (17)	91.5 (43)	0.418
LTRA use, % (*N*)	6.0 (4)	10.0 (2)	4.3 (2)	0.577
INFLAMMATORY PROFILES				
Blood/Serum				
Eosinophil count ≥ 0.2 × 10^9^/L, *% (N*)	61.2 (41)	95.0 (19)	46.8 (22)	**< 0.001**
Neutrophil count, x 10^9^/L	3.88 (1.84)	4.06 (2.68)	3.80 (1.38)	0.608
Aspergillus fumigatus-specific IgE, IU/L * Missing*	0.18 (0.58) 5	0.54 (0.97) 1	0.02 (0.06) 4	**0.001**
Airway				
FeNO ≥ 20 ppb, % (*N*)	61.2 (41)	100.0 (20)	44.7 (21)	**< 0.0001**
Sputum Eosinophils, % * Missing*	5.98 (8.01) 60	13.13 (11.50) 18	3.13 (5.25) 42	0.143
Sputum Neutrophils, % * Missing*	24.09 (23.80) 60	10.63 (0.53) 18	29.48 (26.89) 42	0.381
Sputum Inflammatory Phenotype, *% (N*)				
Eosinophilic Neutrophilic Mixed granulocytic Paucigranulocytic	28.6 (2) 0 (0) 14.3 (1) 57.1 (4)	100.0 (2) 0 (0) 0 (0) 0 (0)	0 (0) 0 (0) 20.0 (1) 80.0 (4)	0.7099
T2 Disease Status				
GINA T2-high status, % (*N*) * Missing*	87.7 (57) 2	100.0 (20) 0	82.2 (37) 2	0.051
COMORBIDITIES *% (N*)				
Rhinitis	83.6 (56)	90.0 (18)	80.9 (38)	0.484
GORD	55.2 (37)	55.0 (11)	55.3 (26)	> 0.999
Obesity (BMI ≥ 30)	31.3 (21)	10.0 (2)	40.4 (19)	**0.020**
Breathing Pattern Disorder	44.8 (30)	30.0 (6)	51.1 (24)	0.179
Inducible Laryngeal Obstruction	6.0 (4)	10.0 (2)	4.3 (2)	0.577
Depression	58.2 (39)	65.0 (13)	55.3 (26)	0.591
Anxiety	56.7 (38)	65.0 (13)	53.2 (25)	0.428
Eczema ever	49.3 (33)	40.0 (8)	53.2 (25)	0.425
Nasal polyps ever	9.1 (6/66)	10.0 (2)	8.7 (4/46)	> 0.999
Salicylate sensitivity ever	7.5 (5)	5.0 (1)	8.5 (4)	> 0.999
Sulphite sensitivity ever	7.5 (5)	5.0 (1)	8.5 (4)	> 0.999
ABPA/SAFS ever	0	0	0	-
Non-CF bronchiectasis ever	0	0	0	-
COPD ever	0	0	0	-
HEALTH AND DISEASE-RELATED QUESTIONNAIRES				
ACQ6 score ≥ 1.50 * Missing*	16.7 (11) 1	31.6 (6) 1	10.6 (5) 0	0.065
SNOT-22 score	21.3 (17.9)	25.4 (21.3)	19.6 (16.2)	0.225
Hull cough hypersensitivity score	11.6 (10.2)	11.5 (11.5)	11.6 (9.7)	0.960
Hull cough hypersensitivity score > 13, % (*N*)	34.3 (23)	25.0 (5)	38.3 (18)	0.402
Nijmegen score	15.1 (10.0)	16.8 (10.3)	14.4 (9.9)	0.375
Nijmegen Score ≥ 23.0, *%* (*N*)	22.4 (15)	25.0 (5)	21.3 (10)	0.756
HADS-D score	2.7 (2.6)	2.6 (2.4)	2.8 (2.7)	0.790
HADS-D Score ≥ 11.0, *%* (*N*)	0	0	0	-
HADS-A score	7.3 (4.2)	7.3 (4.1)	7.3 (4.4)	0.937
HADS-A Score ≥ 11.0, *%* (*N*)	22.4 (15)	15.0 (3)	25.5 (12)	0.524
Equation 5D-5 L Health Today VAS	84.2 (14.7)	84.2 (13.5)	84.3 (15.3)	0.975
SGRQ total score * Missing*	17.7 (13.2) 5	19.1 (14.4) 0	17.0 (12.8) 5	0.560

Categorical variables are shown as % (N). Continuous variables are presented as median (IQR) with minimum and maximum; for normally distributed data, means (SD) are reported. Missing values are indicated. Fisher’s exact test was used for categorical variables; Mann–Whitney U for non-parametric continuous data and unpaired t-test for parametric data. P values <0.05 are considered statistically significant (bolded)

*Abbreviations: GP *general practice, *OCS *oral corticosteroids, *ICU *Intensive Care Unit, *FeNO *fractional exhaled nitric oxide, *ppb *parts per billion, *IgE *immunoglobulin E, *BD *bronchodilator, *FEV₁ *forced expiratory volume in 1 second, *SPT *skin prick test, *ACQ6 *Asthma Control Questionnaire-6, *BDP equiv. *beclomethasone dipropionate equivalent/day, *SABA *short-acting β2-agonist, *LTRA *leukotriene receptor antagonist, *T2 *type-2, *GINA *Global Initiative for Asthma, *GORD *gastro-oesophageal disease, *ABPA/SAFS *allergic bronchopulmonary aspergillosis/severe asthma with fungal sensitisation, *CF *cystic fibrosis, *COPD *chronic obstructive pulmonary disease, *SNOT-22 *Sino-Nasal Outcome Test-22, *HADS-D *Hospital Anxiety and Depression scale-Depression component, *HADS-A *Hospital Anxiety and Depression scale-Anxiety component, *EQ5D-5L Health Today VAS *EuroQoL 5-Dimension 5-Level Health Today Visual Analogue Scale, *SGRQ *St Georges Respiratory Questionnaire

- Cluster-1 (*n* = 20; 29.9%); *Early-onset atopic mild*: early childhood-onset disease with raised FeNO, BEC and total IgE, greater atopy, lower FEV₁ % predicted and higher ACQ-6.
- Cluster-2 (*n* = 47; 70.1%); *Adolescent-onset mild*: adolescent-onset disease with lower inflammatory biomarker expression and atopy, better FEV₁ % predicted and lower ACQ-6.

#### Post-hoc cluster characterisation

Summary characterisation of the mild asthma clustering variables, baseline clinical characteristics, inflammatory profiles, comorbidities, health-related quality of life and disease-related questionnaire outcomes is shown in Table 4. Lung function measurements are presented in Supplementary Table 3.

- Cluster-1: Early-onset atopic mild. This cluster showed male predominance and early childhood-onset asthma. It had a stronger T2 profile, with higher FeNO, BEC, total IgE and Aspergillus fumigatus-specific IgE, greater atopic burden, and universal GINA-defined T2-high status. Lung function was preserved overall, but pre-BD FEV₁ % predicted, FEV₁/FVC ratio and FEF₂₅–₇₅ were lower than in cluster-2. ACQ-6 was higher, indicating relatively worse asthma control within the mild asthma cohort. Comorbidities were common, although broadly similar to cluster-2.
- Cluster-2: *Adolescent-onset mild*. This larger cluster showed female predominance and adolescent-onset asthma. It had lower FeNO, BEC, total IgE, Aspergillus fumigatus-specific IgE and atopic burden than cluster-1, although GINA-defined T2-high status remained common. Lung function was preserved and was modestly better than in cluster-1, with higher pre-BD FEV₁ % predicted, FEV₁/FVC ratio and FEF₂₅–₇₅. ACQ-6 was lower, indicating better asthma control. Obesity was more prevalent in this cluster, while other comorbidities and questionnaire outcomes were broadly similar between the two mild asthma clusters.

### Summary characterisation of difficult-to-treat and mild asthma cluster phenotypes

A summary of the six DA and two MA cluster phenotypes respectively, incorporating clustering and morbidity characteristics is shown in Fig. 1.

## Discussion

Using a common assessment platform across two cohorts spanning the asthma severity spectrum, we identified six difficult-to-treat clusters and two mild-asthma clusters with distinct clinical, inflammatory and comorbidity profiles. A key novel focus of this study was the exploration of comorbidities and highlighting their role in asthma heterogeneity. One important finding is that despite using an identical phenotyping platform for comprehensive clinical characterisation, heterogeneity is already apparent in milder asthma and does not map directly onto patterns seen in more difficult-to-treat disease. Separate K-means clustering analyses for the WATCH-DA and EOSA-MA cohorts using identical clinical and biomarker assessments revealed distinct phenotypic profiles across both groups. Although T2-high biology was common, it did not alone explain variation in symptoms, physiology, or multimorbidity. Among difficult-to-treat clusters, disease severity, sex, age of onset, and BMI varied alongside airflow limitation and healthcare use. The mild asthma clusters, despite preserved spirometry, were clinically and biologically distinct with significant comorbidities suggesting multimorbid burden.

Comparisons across asthma severities indicate that burden is shaped by combinations of pulmonary and extra-pulmonary traits rather than a single linear progression from mild to difficult-to-treat disease. This perspective clarifies how the phenotypes relate across the disease spectrum and provides a practical basis for trait-targeted, guideline-aligned care.

Our results reflect and extend existing literature showing reproducible, though not identical, asthma clinical phenotypes, including early-onset atopic T2-high, adult-onset eosinophilic, and obese difficult asthma [8, 9, 11]. While the cluster characteristics inevitably depend on the clinical and pathobiological parameters collected, these studies consistently demonstrate that clinically recognisable clusters can share T2 features yet diverge in physiology and symptom burden, emphasising the need for multi-domain appraisal rather than reliance on T2-biomarkers alone. Large population and longitudinal cohorts further underscore the breadth of multimorbidity across asthma severities, with adult-onset disease and obesity, particularly among women, associated with greater morbidity over time [26, 27]. While our cross-sectional design precludes inference about directionality, the prominence of comorbidities in both the difficult-to-treat and mild asthma cohorts is concordant with these observations and merits routine clinical attention.

Obesity, which has long reached epidemic proportions, warrants particular discussion. In our difficult-to-treat clusters, higher BMI co-occurred with greater symptom burden and lower T2 signals in subsets, while in mild asthma a lower-T2, obesity-linked group exhibited good spirometry but notable patient-reported burden. Observational and intervention studies suggest that weight loss can be associated with improvements in asthma control and quality of life in individuals with obesity; however, responses vary and should be considered alongside optimisation of inhaled therapy and comorbidity management [26–29]. We therefore interpret obesity as a clinically relevant trait that frequently co-occurs with asthma across the asthma severity spectrum.

Sex and age of onset patterns were also informative. Prior work has described female predominance in obesity-linked phenotypes and higher risk associated with adult-onset disease; our clusters showed similar associations [8, 9, 11, 27]. These readily available cues: sex, age of onset, and BMI, remain practical anchors for recognising phenotypes, particularly when considered alongside simple T2-biomarkers, basic spirometry and a focused review of extra-pulmonary traits.

Comparison across both cohorts showed that one mild asthma cluster exhibited greater morbidity but did not map one-to-one onto any single difficult-to-treat asthma cluster. Instead, overlapping mixtures of traits were seen across severities, supporting the view that heterogeneity is also present in upstream milder disease and amplifies with accrued comorbidity regardless of asthma severity. This observation argues for early identification and management of co-existing traits between mild and difficult-to-treat clusters even when spirometry is near-normal.

Our findings support the clinical relevance of multi-domain asthma phenotyping, particularly where symptom burden, exacerbation risk, airflow obstruction and comorbidities do not align with T2 biomarkers alone. However, the present study was observational and cross-sectional, and treatments were not assigned or tested according to cluster membership. Therefore, the clusters should not be interpreted as treatment algorithms. Instead, they provide clinically recognisable profiles that may help structure assessment, highlight co-existing treatable traits, and generate hypotheses for future prospective studies evaluating whether cluster-informed management improves outcomes beyond current guideline-based care.

Our study has both strengths and limitations. Strengths include harmonised clustering across two well-phenotyped cohorts which have been identically characterised. Use of clinically relevant clustering variables adds further robustness. Also, deep post-hoc characterisation spanning inflammatory, physiology, comorbidities and patient-reported outcomes/HRQoL assessments of both mild and difficult-to-treat asthma patients. One potential limitation is the cross-sectional design, and future studies would benefit from assessing longitudinal cluster stability. Limited induced sputum and airway sampling, particularly in the MA cohort, may be viewed as another shortcoming. Nevertheless, an adequate proportion of airway sampling was achieved in the WATCH-DA cohort permitting valid inferences. While the study might be viewed as single centre, the WATCH-DA cohort is drawn from a tertiary referral site that effectively serves as a multi-centre checkpoint. These considerations do not alter the central practice message that comorbidity-linked treatable traits drive multimorbid burden across phenotypes.

The EOSA-MA cohort was small compared with the WATCH-DA cohort, which limited statistical power and the resolution of clustering in mild asthma. Consequently, only a two-cluster solution could be robustly supported, and more subtle mild asthma phenotypes may have been missed. These findings should therefore be considered exploratory and require replication in larger mild asthma populations.

Future steps include using these clusters to validate a minimal clinical recognition algorithm (age of onset, BMI, atopy, FeNO/BEC plus 2–3 comorbidity traits) against patient outcomes and treatment response; to assess phenotype-tailored therapeutic interventions; to prospectively evaluate OCS-sparing strategies in high-burden groups; and integrate omics/imaging where comorbidity signals are strongest (obesity, CRSwNP, AERD, ABPA/SAFS), incorporating sex-specific analyses.

In summary, across difficult-to-treat and mild asthma cohorts assessed with a comparable clinical characterisation platform, clustering reveals clinically coherent associations between biomarker patterns, physiological measures and multimorbidity on a largely T2-predominant background. Used cautiously in alignment with guidelines and trait-targeted evidence, these patterns can help clinicians recognise asthma phenotypes rapidly and instigate first-line interventions earlier for active treatable traits.

## Supplementary Information

Supplementary Material 1.

## Data Availability

The datasets generated and/or analysed during the current study are not publicly available due to participant confidentiality and governance restrictions associated with the WATCH-DA and EOSA cohorts but are available from the corresponding author on reasonable request.

## Abbreviations

ABPA: Allergic bronchopulmonary aspergillosis
ACQ6: Asthma Control Questionnaire-6
BD: Bronchodilator
BEC: Blood eosinophil count
BMI: Body Mass Index
BN: Blood neutrophils count
BTS: British Thoracic Society
CCC: Cubic Clustering Criterion
COPD: Chronic Obstructive Pulmonary Disease
CRSwNP: Chronic rhinosinusitis with nasal polyps
DA: Difficult Asthma
DHAAC: David Hide Asthma and Allergy Centre
EOSA: Epigenetics of Severe Asthma
EQ-5D-5L VAS: EuroQol 5-Dimension 5-Level health today Visual Analogue Scale
FEF _25%-75%_: Forced expiratory flow between 25% and 75% of vital capacity
FeNO: Fractional exhaled nitric oxide
FEV₁: Forced Expiratory Volume in 1 s
FVC: Forced Vital Capacity
GORD: Gastro-oesophageal reflux disease
GINA: Global Initiative for Asthma
GP: General practice
HADS: Hospital Anxiety and Depression Scale
ICU: Intensive Care Unit
IgE: Immunoglobulin E
IOWBC: Isle of Wight Birth Cohort
MA: Mild asthma
NICE: National Institute for Health and Care Excellence
OCS: Oral corticosteroids
QoL: Quality of life
SAFS: Severe asthma with fungal sensitisation
SGRQ: St George’s Respiratory Questionnaire
SNOT-22: Sino-Nasal Outcome Test
SPT: Skin prick testing
T2: Type-2 inflammation
WATCH: Wessex AsThma CoHort of Difficult Asthma

## References

[1] Global Initiative for Asthma. Global Strategy for Asthma Management and Prevention. 2022. https://ginasthma.org/wp-content/uploads/2022/07/GINA-Main-Report-2022-FINAL-22-07-01-WMS.pdf. Accessed 26 May 2026.

[2] Global Initiative for Asthma. Difficult-to-treat & severe asthma in adolescent and adult patients: diagnosis and management. A GINA Pocket Guide for Health Professionals. Version 2.0. 2019. https://ginasthma.org/wp-content/uploads/2019/04/GINA-Severe-asthma-Pocket-Guide-v2.0-wms-1.pdf. Accessed 26 May 2026.

[3] British Thoracic Society; Scottish Intercollegiate Guidelines Network. British guideline on the management of asthma: a national clinical guideline. SIGN 153. Revised September 2016. Edinburgh: Scottish Intercollegiate Guidelines Network; 2016. https://www.sign.ac.uk/guidelines/british-guideline-on-the-management-of-asthma/. Accessed 26 May 2026.

[4] Wenzel SE. Asthma phenotypes: the evolution from clinical to molecular approaches. Nat Med. 2012;18(5):716–25. 22561835 10.1038/nm.2678

[5] Chung KF. Precision medicine in asthma: linking phenotypes to targeted treatments. Curr Opin Pulm Med. 2018;24(1):4–10. 29036018 10.1097/MCP.0000000000000434

[6] Lötvall J, Akdis CA, Bacharier LB. Asthma endotypes: a new approach to classification of disease entities within the asthma syndrome. J Allergy Clin Immunol. 2011;127(2):355–60. 21281866 10.1016/j.jaci.2010.11.037

[7] Kuruvilla ME, Lee FE, Lee GB. Understanding asthma phenotypes, endotypes, and mechanisms of disease. Clin Rev Allergy Immunol. 2019;56(2):219–33. 30206782 10.1007/s12016-018-8712-1 PMC6411459

[8] Haldar P, Pavord ID, Shaw DE. Cluster analysis and clinical asthma phenotypes. Am J Respir Crit Care Med. 2008;178(3):218–24. 18480428 10.1164/rccm.200711-1754OC PMC3992366

[9] Lefaudeux D, De Meulder B, Loza MJ. U-BIOPRED clinical adult asthma clusters linked to a subset of sputum omics. J Allergy Clin Immunol. 2017;139(6):1797–807. 27773852 10.1016/j.jaci.2016.08.048

[10] Newby C, Heaney LG, Menzies-Gow A. Statistical cluster analysis of the British Thoracic Society Severe Refractory Asthma Registry: clinical outcomes and phenotype stability. PLoS ONE. 2014;9(7):e102987. 25058007 10.1371/journal.pone.0102987 PMC4109965

[11] Moore WC, Meyers DA, Wenzel SE. Identification of asthma phenotypes using cluster analysis in the Severe Asthma Research Program. Am J Respir Crit Care Med. 2010;181(4):315–23. 19892860 10.1164/rccm.200906-0896OC PMC2822971

[12] Fitzpatrick AM, Teague WG, Meyers DA, et al. Heterogeneity of severe asthma in childhood: confirmation by cluster analysis of children in the National Institutes of Health/National Heart, Lung, and Blood Institute Severe Asthma Research Program. J Allergy Clin Immunol. 2011;127(2):382–e91. 21195471 10.1016/j.jaci.2010.11.015 PMC3060668

[13] Fitzpatrick AM, Moore WC. Severe asthma phenotypes - how should they guide evaluation and treatment? J Allergy Clin Immunol Pract. 2017;5(4):901–8. 28689840 10.1016/j.jaip.2017.05.015 PMC5541906

[14] Bal C, Pohl W, Milger K, et al. Characterization of obesity in severe asthma in the german asthma net. J Allergy Clin Immunol Pract. 2023;11(11):3417–e243. 37406803 10.1016/j.jaip.2023.06.049

[15] Tariq K, Schofield JPR, Nicholas BL, et al. Sputum proteomic signature of gastro-oesophageal reflux in patients with severe asthma. Respir Med. 2019;150:66–73. 30961953 10.1016/j.rmed.2019.02.008

[16] Langdon C, Mullol J. Nasal polyps in patients with asthma: prevalence, impact, and management challenges. J Asthma Allergy. 2016;9:45–53. 27042129 10.2147/JAA.S86251 PMC4798207

[17] Freeman A, Abraham S, Kadalayil L, et al. Associations of breathing pattern disorder and Nijmegen score with clinical outcomes in difficult-to-treat asthma. J Allergy Clin Immunol Pract. 2024;12(4):938–e476. 38036249 10.1016/j.jaip.2023.11.036

[18] Fong WCG, Rafiq I, Harvey M, Stanescu S, Ainsworth B, Varkonyi-Sepp J, et al. The detrimental clinical associations of anxiety and depression with difficult asthma outcomes. J Pers Med. 2022;12(5):686. 10.3390/jpm12050686. 10.3390/jpm12050686 PMC9142921 35629109

[19] Scelo G, Torres-Duque CA, Maspero J, et al. Analysis of comorbidities and multimorbidity in adult patients in the International Severe Asthma Registry. Ann Allergy Asthma Immunol. 2024;132(1):42–53. 37640263 10.1016/j.anai.2023.08.021

[20] Shackleford A, Heaney LG, Redmond C, McDowell PJ, Busby J. Clinical remission attainment, definitions, and correlates among patients with severe asthma treated with biologics: a systematic review and meta-analysis. Lancet Respir Med. 2025;13(1):23–34. 39549709 10.1016/S2213-2600(24)00293-5

[21] Kurukulaaratchy RJ, Freeman A, Bansal AT, et al. Evaluation of the effect of multimorbidity on difficult-to-treat asthma using a novel score (MiDAS): a multinational study of asthma cohorts. Lancet Respir Med. 2025;13(9):821–32. 40645203 10.1016/S2213-2600(25)00135-3

[22] Azim A, Mistry H, Freeman A, Barber C, Newell C, Gove K, et al. Protocol for the Wessex AsThma CoHort of difficult asthma (WATCH): a pragmatic real-life longitudinal study of difficult asthma in the clinic. BMC Pulm Med. 2019;19(1):99. 10.1186/s12890-019-0862-2 PMC6534885 31126281

[23] Arshad SH, Patil V, Mitchell F. Cohort profile update: the Isle of Wight whole population birth cohort (IOWBC). Int J Epidemiol. 2020;49(4):1083–4. 32637984 10.1093/ije/dyaa068 PMC7660140

[24] Global Initiative for Asthma. Global Strategy for Asthma Management and Prevention. 2016. https://ginasthma.org/wp-content/uploads/2016/04/GINA-2016-main-report_tracked.pdf. Accessed 26 May 2026. 37865091 10.1016/j.medj.2023.09.003 PMC10964988

[25] Herrera-De La Mata S, Ramíírez-Suáástegui C, Mistry H, et al. Cytotoxic CD4(+) tissue-resident memory T cells are associated with asthma severity. Med. 2023;4(12):875–97.e8. 37865091 10.1016/j.medj.2023.09.003 PMC10964988

[26] Kankaanranta H, Viinanen A,Ilmarinen P, et al. Comorbidity burden in severe and nonsevere asthma: a nationwide observational study (FINASTHMA). J Allergy Clin Immunol Pract. 2024;12(1):135–45.e9. 37797715 10.1016/j.jaip.2023.09.034

[27] Backman H, Stridsman C, Hedman L, et al. Determinants of severe asthma: a long-term cohort study in Northern Sweden. J Asthma Allergy. 2022;15:1429–39. 36248343 10.2147/JAA.S376806 PMC9562796

[28] Scott HA, Gibson PG, Garg ML, et al. Dietary restriction and exercise improve airway inflammation and clinical outcomes in overweight and obese asthma: a randomized trial. Clin Exp Allergy. 2013;43(1):36–49. 23278879 10.1111/cea.12004

[29] Freitas PD, Ferreira PG, Silva AG, et al. The role of exercise in a weight-loss program on clinical control in obese adults with asthma: a randomized controlled trial. Am J Respir Crit Care Med. 2017;195(1):32–42. 27744739 10.1164/rccm.201603-0446OC

