# Supplementary material for: Clinical phenotypes of difficult-to-treat and mild asthma defined by cluster analysis
Source: Respir Res. 2026 May 29;27:306. doi: 10.1186/s12931-026-03740-0 (PMC13480206; doi:10.1186/s12931-026-03740-0)
Supplement: Supplementary file 1 [file 12931_2026_3740_MOESM1_ESM.docx]

**Supplementary Materials**

**Supplementary Figure 1:**

Supplementary Figure 1: Participant flow for the WATCH difficult asthma and NIH EOSA mild asthma cohorts

The diagram shows recruitment sources, exclusions, and final analytic samples for the two independently analysed UK cohorts.
Left (WATCH): Adults with difficult asthma were enrolled from the University Hospital Southampton NHS Foundation Trust (UHSFT) Regional Difficult Asthma Clinics and the David Hide Asthma & Allergy Research Centre (DHAARC), Isle of Wight (IOW) (n=501; July 2015–March 2019). Three participants were excluded as extreme outliers for total serum IgE, yielding n=498 for clustering.
Right (NIH EOSA): Adults with mild asthma (BTS/SIGN steps 1–2; GINA steps 1–2) were recruited August 2018–July 2019 from two sources: the Isle of Wight Birth Cohort (IOWBC) (n=23) and local IOW allergy clinics/primary care (n=46), forming the NIH EOSA mild asthma cohort (n=69). Two were excluded (one heavy smoking; one extreme raised total serum IgE), yielding n=67 for clustering.

# **Supplementary Table 1: Comorbidity Definitions and Operationalisation (WATCH and NIH EOSA)**

| Comorbidity | Operational definition used in this study | Notes / instruments |
| --- | --- | --- |
| Rhinitis (allergic/non‑allergic) | Clinician-diagnosed rhinitis recorded in the medical record (ever/current). | Allergic status supported by SPT where relevant; not required for coding. |
| Gastro‑oesophageal reflux disease (GORD/GERD) | Clinician-diagnosed GORD documented in the record and/or ongoing anti‑reflux therapy. | Symptom-based diagnosis in routine care; endoscopy where available. |
| Obesity | Body mass index (BMI) ≥ 30 kg/m² measured at enrolment. | Categorical variable used in analyses. |
| Breathing pattern disorder ("dysfunctional breathing") | Clinician-diagnosed breathing pattern disorder (physiotherapy/respiratory specialist assessment). | Screening questionnaire: Nijmegen score; threshold ≥23 indicates positive screen. |
| Intermittent laryngeal dysfunction (inducible laryngeal obstruction) | Specialist-diagnosed vocal cord/inducible laryngeal dysfunction recorded in the notes (ever). | ENT/SLT assessment where available. |
| Depression | Clinician-diagnosed depression recorded in the medical record (ever). | Screening instrument: HADS‑D; score ≥11 indicates probable case (reported separately). |
| Anxiety | Clinician-diagnosed anxiety disorder recorded in the medical record (ever). | Screening instrument: HADS‑A; score ≥11 indicates probable case (reported separately). |
| Eczema (atopic dermatitis) | Clinician-diagnosed eczema recorded in the medical record (ever). | — |
| Nasal polyps | Clinician/ENT-diagnosed nasal polyps recorded in the medical record (ever). | Often supported by Nasoendoscopy or CT; CRSwNP considered within this category. |
| Salicylate (aspirin/NSAID) sensitivity | History consistent with aspirin/NSAID‑exacerbated respiratory symptoms recorded by a clinician (ever). | Relates to AERD spectrum; formal provocation testing not required for coding. |
| Sulphite sensitivity | History of sulphite‑related respiratory symptoms recorded by a clinician (ever). | — |
| Allergic bronchopulmonary aspergillosis (ABPA) / Severe asthma with fungal sensitisation (SAFS) | Specialist diagnosis of ABPA and/or SAFS recorded in the medical record (ever). | ABPA typically per standard criteria (elevated total/specific IgE, radiology); SAFS = severe asthma plus fungal sensitisation without ABPA features. |
| Non‑CF bronchiectasis | Radiologically confirmed bronchiectasis on HRCT recorded in the medical record (ever). | Cystic fibrosis excluded. |
| Chronic obstructive pulmonary disease (COPD) | Clinician-diagnosed COPD recorded in the medical record (ever). | Typically supported by persistent post‑bronchodilator airflow obstruction and exposure history. |

Notes: Comorbidities were captured from clinical records and coded using conventional clinical criteria. Questionnaire-based screening thresholds are shown where applicable.

**Supplementary Methodology**

*Variable Selection for Clustering Analysis*

Clinical variables used for cluster analysis were selected based on consensus drawn from reviewing the literature for previous clustering analysis^8-11^ and those of clinical relevance in the evaluation of asthma including:

a) clinically important disease- and pathobiological-defining variables reflecting asthma severity, symptom control and objective physiological and biomarker parameters,

b) variables that were easy to measure and common in clinical practice

c) exclusion of variables that may bias and influence identified phenotypes e.g. sex, current age and comorbidities, or which showed collinearity to other cluster variables.

**Supplementary Table 2: Lung function test measurements for the WATCH difficult asthma clusters**

| **Lung Function Tests** | **Total Cohort** | **Cluster 1** | **Cluster 2** | **Cluster 3** | **Cluster 4** | **Cluster 5** | **Cluster 6** | ***P*-value** |
| --- | --- | --- | --- | --- | --- | --- | --- | --- |
| **Clinic (Post-BD) Spirometry, *% (N)***  Post-BD FEV_1_, % pred. | 75.5 (22.9) | **78.4** (22.2) | 75.1 (16.4) | ***61.9*** (21.6) | 76.5 (23.8) | 70.5 (28.0) | 76.6 (21.6) | **0.030** |
| *Missing* | 160 | 57 | 15 | 10 | 37 | 7 | 34 |  |
| Post-BD FVC, % pred. | 90.3 (18.4) | 91.8 (17.1) | **98.1** (19.9) | ***82.6*** (16.2) | 92.7 (19.1) | 85.2 (18.5) | 85.5 (18.1) | **0.004** |
| *Missing* | 161 | 57 | 15 | 10 | 37 | 8 | 34 |  |
| Post-BD FEV_1_/FVC Ratio, % | 66.3 (14.7) | 68.3 (14.5) | 61.8 (11.0) | ***59.7*** (16.0) | 63.9 (14.9) | 64.6 (18.4) | **71.7** (11.4) | **0.001** |
| *Missing* | 160 | 56 | 15 | 10 | 37 | 8 | 34 |  |
| Post-BD FEF 25-75%, % pred. | 55.7 (35.9) | 59.4 (35.7) | 42.7 (22.8) | ***35.9*** (25.8) | 57.3 (39.7) | 50.8 (42.1) | **62.5** (31.6) | **0.010** |
| *Missing* | 160 | 57 | 15 | 10 | 37 | 7 | 34 |  |
| **Lung Volumes**  Forced Residual Capacity, % pred. | 100.5 (25.3) | 97.6 (20.3) | 87.1 (16.0) | 98.0 (13.2) | 102.3 (28.4) | 110.2 (27.5) | 100.4 (34.5) | 0.795 |
| *Missing* | 380 | 129 | 30 | 30 | 97 | 22 | 72 |  |
| Total Lung Capacity, % pred. | 102.8 (14.2) | 103.9 (14.4) | 102.0 (7.2) | 94.8 (18.9) | 102.2 (15.2) | 101.8 (10.8) | 104.6 (15.6) | 0.813 |
| *Missing* | 379 | 129 | 30 | 30 | 96 | 22 | 72 |  |
| Vital Capacity, % pred. | 92.1 (19.3) | 93.8 (20.2) | 92.4 (18.1) | 78.0 (22.8) | 98.0 (15.7) | 85.2 (9.2) | 87.3 (23.8) | 0.201 |
| *Missing* | 392 | 133 | 30 | 31 | 102 | 23 | 73 |  |
| Residual Volume, % pred. | 120.5 (38.9) | 116.4 (31.2) | 114.4 (33.0) | 123.6 (28.1) | 112.3 (35.1) | 142.5 (51.2) | 131.7 (51.8) | 0.201 |
| *Missing* | 380 | 129 | 30 | 30 | 97 | 22 | 72 |  |
| RV/TLC Ratio, % | 39.7 (11.2) | ***36.1*** (10.4) | 37.2 (11.7) | **49.4** (9.3) | 39.6 (8.7) | 45.2 (10.0) | 43.0 (14.2) | **0.021** |
| *Missing* | 380 | 129 | 30 | 30 | 97 | 22 | 72 |  |
| **Gas Transfer Factor**  TLco Hb, % pred. | 88.3 (18.2) | 90.8 (15.6) | 95.9 (11.6) | 82.3 (22.9) | 85.8 (21.3) | 84.9 (19.0) | 86.8 (18.1) | 0.188 |
| *Missing* | 301 | 101 | 23 | 20 | 82 | 17 | 58 |  |
| Kco Hb, % pred. | 99.8 (17.9) | 98.7 (15.0) | 100.5 (14.0) | 108.7 (21.6) | 97.2 (21.4) | 99.7 (17.9) | 101.4 (18.5) | 0.395 |
| *Missing* | 301 | 101 | 23 | 20 | 82 | 17 | 58 |  |

Notes: Continuous variables are represented as mean (SD). The number of missing values is included. One-way ANOVA was applied to derive significance values between the 6 clusters. P-values ,0.05 are significant (in bold). Significantly different clusters are highlighted in bold (highest) and bold italics (lowest). Abbreviations: BD, bronchodilator; FEV1, forced expiratory volume in 1 second; % pred., % predicted; FVC, forced vital capacity; FEF 25-75%, forced expiratory flow between 25-75% exhalation; RV/TLC, residual volume/total lung capacity; TLco Hb, transfer factor of the lung for carbon monoxide adjusted for haemoglobin concentration; Kco Hb, carbon monoxide transfer coefficient adjusted for haemoglobin concentration.

**Supplementary Table 3. Lung function test measurements of the EOSA mild asthma clusters**

| **Lung Function Tests** | **Total Cohort** | **Cluster 1** | **Cluster 2** | ***P*-value** |
| --- | --- | --- | --- | --- |
| Pre-BD FEV_1_, % pred.  Mean (SD)  Min, Max | 91.4 (14.5)  62.0, 138.0 | 84.8 (18.0)  62.0, 138.0 | 94.4 (11.8)  63.0, 117.0 | **0.006** ^#^ |
| Pre-BD FVC, % pred.  Mean (SD)  Min, Max | 100.2 (12.6)  71.0, 129.0 | 96.7 (13.5)  79.0, 129.0 | 101.3 (12.3)  71.0, 129.0 | 0.182 ^Ɨ^ |
| Pre-BD FEV_1_/FVC Ratio, %  Mean (SD)  Min, Max | 74.8 (8.5)  56.0, 90.0 | 71.0 (7.9)  57.0, 83.0 | 76.9 (7.8)  60.0, 90.0 | **0.007** ^Ɨ^ |
| Pre-BD FEF 25-75%, % pred.  Median (IQR)  Min, Max | 74.0 (38.0)  35.0, 174.0 | 57.0 (35.8)  35.0, 174.0 | 85.0 (37.0)  38.0, 148.0 | **0.001** ^#^ |

Notes. Continuous variables are represented as median and interquartile range (IQR) for non-parametric data and mean (SD) for parametric data. Minimum (min) and maximum (max) values are shown. Mann Whitney U test (#) was applied for non-parametric continuous variables and unpaired t-test (Ɨ) for parametric data. P-values <0.05 are significant (in bold). Abbreviations: BD, bronchodilator; FEV_1_, forced expiratory volume in 1 second; % pred., % predicted; FVC, forced vital capacity; FEF 25-75%, forced expiratory flow between 25-75% exhalation.
